# Predictors of participation in preventive health examinations in Austria

**DOI:** 10.1186/1471-2458-13-1138

**Published:** 2013-12-05

**Authors:** Sophie Brunner-Ziegler, Anita Rieder, Katharina Viktoria Stein, Renate Koppensteiner, Kathryn Hoffmann, Thomas Ernst Dorner

**Affiliations:** 1 Institute of Social Medicine, Center for Public Health, Medical University of Vienna, Kinderspitalgasse 15/I, Vienna, Austria; 2 Department for Internal Medicine, Division of Angiology, Medical University of Vienna, Währinger Gürtel 18-20, Vienna, Austria; 3 Department of General Practice and Family Medicine, Center for Public Health, Medical University of Vienna, Kinderspitalgasse 15/I, Vienna, Austria

**Keywords:** Preventive health services, Socio-economic status, Chronic diseases, Subjective health status

## Abstract

**Background:**

Preventive health check-ups in Austria are offered free of charge to all insured adults (98% of the population) and focus on early detection of chronic diseases, primary prevention, and health counseling. The study aims to explore predictors of compliance with the recommended interval of preventive health check-up performance.

**Methods:**

Source of data was the Austrian Health Interview Survey 2006/07 (15,474 subjects). Participation in a preventive health examination during the last three years was used as dependent variable. Socio-demographic and health-related characteristics were used as independent variables in a multivariate logistic regression analysis.

**Results:**

Results show that 41.6% of men and 41.8% of women had attended a preventive health check-up within the last three years. In multivariate analysis, subjects ≥40 years, with higher education, higher income or born in Austria were significantly more likely to attend a preventive health check-up. Furthermore, a chronic disease was associated with a higher attendance rate (OR: 1.21; CI: 1.07-1.36 in men; OR: 1.19; CI: 1.06-1.33 in women).

**Conclusions:**

Attendance rates for health check-ups in the general Austrian population are comparatively high but not equally distributed among subgroups. Health check-ups must increase among people at a young age, with a lower socio-economic status, migration background and in good health.

## Background

In view of the steady increase of life expectancy and the corresponding rise of chronic disease rates during the past decade, health promotion and primary and secondary preventive health services have become more and more important [1]. While secondary preventive strategies, such as cancer screening programs are designed for the early detection of a disease, primary preventive measures aim to avoid the occurrence of a disease and include immunization programs, assessments of cardiovascular risk factors and occupational-related general health check-ups. All kinds of preventive health measures positively influence health behavior intentionally or as a side effect [2].

Most studies on preventive health check-ups were carried out in minority subpopulations of countries with large socio-demographic disparities, in which access to health care is not equally available to all [3]. However, data from economically well established countries is rarely available. Countries in the Organization for Economic Co-operation and Development have the most developed national guidelines or recommendations for the implementation of health promotion measures and preventive health examination programs. Such programs have already successfully been established in most northern states such as Sweden and Great Britain, whereas, in comparison, many southern states, such as Greece and Italy, lack health promotion and preventive health initiatives [4].

Austria’s social security system is built on the principle of solidarity and equality. Fees for health insurance are related to the individual income but all health care services are equally offered to everyone. Every person as of the age of 19 and living in Austria is entitled to an annual preventive health check-up, which is free of charge. This service is composed of a comprehensive program, focused on prevention and early detection of diseases, including cardiovascular and metabolic risk factors and cancer (such as breast cancer, cervical cancer and colon cancer), prevention of addictive disorders (such as tobacco or alcohol dependency), periodontal diseases and older-age-related diseases (such as disorders of the eyes and ears). Basic examinations include blood samples on the first day, a physical examination, an examination of the health behavior, a risk factor evaluation and a consolatory debriefing on another day. They can either go to a family physician or a specialist for internal medicine with a valid sickness fund or in a national health clinic. The expenses are reimbursed on an equal basis at slightly higher rates than usually provided for medical consultations. Patients are referred to as specialists for respective complementary examinations, such as mammography, colonoscopy and PAP-smear. The offer of complementary examinations is stratified by gender and age (i.e. offer of colonoscopy at the age of 50, of examination of the eyes and ears at the age of 65,…). Fifty percent of the Austrian population has had at least one comprehensive preventive health check-up. Public health services though recommend attending preventive health reassessments on a regular basis for people of all age groups and propose that elderly people should attend a health check-up even more often because of the correlation between age and health related complaints [5].

An international comparison of general attendance rates at routinely offered preventive health services is difficult as preventive and screening examinations are defined differently in various health care systems. Most recently, the US Preventive Service Task Force has questioned the long-term benefit of periodic comprehensive preventive health examinations on morbidity and mortality. Instead, an incorporation of specific individual preventive health services has been recommended [6]. Understanding the principle determinants in preventive health practices is of major importance in order to optimize any kind of preventive health strategy. For breast cancer screening and also colorectal cancer screening various factors are said to influence women’s decision on doing a health check-up, including characteristics of the patient, the health care providers and the environment [7]. In addition, patients without health insurance were less likely to have a cancer screening and attend counseling for preventive health services [8].

The aim of the present study was to explore socio-economic and health status-related characteristics and predictors for compliance with preventive health check-up performance recommendations in the Austrian population.

## Methods

Data analyzed for the present study were derived from the Austrian Health Interview Survey database (AT-HIS) 2006–07 [5]. This survey, based on the European Core Health Interview Survey and adapted to Austrian conditions, was commissioned by the Austrian Federal Ministry of Health, Family and Youth and carried out by Statistics Austria [5]. The interviews were conducted face-to-face using computer assisted personal interviewing. A final sample of 15,474 subjects aged 15 years and older (out of a total of 25,130 candidates) were included for analysis, resulting in a response rate of 63.1%. Prior investigations by Statistics Austria showed that within the Austrian population response rates to surveys are generally equally distributed among all age groups and geographic regions [9]. For the present study, only subjects aged 20 years or older were included, thus the final sample consisted of 6,982 men and 7,487 women. The sample was weighted according to geographic region, age, and sex, to account for the stratification. Besides evaluation of socio-demographic and socio-economic parameters the 450 questions surveyed information on the presence of diseases and/or health complaints, the subjective health status and quality of life, the individual health behavior and the utilization of health care services.

### Study variables

The primary outcome variable and dependent variable in regression analysis was having attended a comprehensive preventive health check-up within the three years preceding the survey with the answer categories “yes” or “no”.

Independent variables in the analysis were the socio-demographic characteristics sex, age, highest achieved educational level, monthly net household income per household member and country of birth. In Austria, rates of migration always have been high. Therefore, the country of birth has been included in the regression model, as habitants, born abroad and/or without the Austrian nationality are characterized by integration with respect to education, career opportunity and also health outcome.

Furthermore, self-rated overall health status and health satisfaction, presence of chronic diseases, body mass index (BMI), and impaired mental health status (subjective perceived psycho-social discomfort) were included in the analysis.

Age was stratified in three group intervals, 1) 20 to 39 years, 2) 40 to 64 years and 3) 65 years and older.

Educational status was categorized as 1) primary education (up to the age of 15 years), 2) secondary education (apprenticeship or secondary school) and 3) tertiary education (university or any further education).

Monthly net household income per household member was divided into three categories, 1) equal or less than 600 Euro, 2) 601 to 1,500 Euro and 3) more than 1,500 Euro.

The country of birth was grouped into five categories 1) born in Austria, 2) born in the European Union (EU)-15, except Austria, or the European Free Trade Association-countries, 3) born in one of the twelve “new” EU-states, 4) born in Turkey or in former Yugoslavian states except Slovenia and 5) born elsewhere.

Self-rated overall health status was assessed in five categories according to the answer to the question “How do you rate your health in general?”, 1) very good, 2) good, 3) moderate, 4) poor and 5) very poor.

Self-rated health satisfaction was classified as 1) feeling satisfied or 2) feeling not satisfied.

The variable “chronic diseases” included the presence of at least one of the following disorders: allergic or other type of asthma bronchiale, allergy, diabetes mellitus, eye cataract, tinnitus, elevation of arterial blood pressure, history of myocardial infarction, history of stroke or cerebral hemorrhage, chronic obstructive pulmonary disease or emphysema, arthrosis or arthritis or rheumatism, chronic back pain, osteoporosis, urinary incontinence, gastro-intestinal ulcer, cancer, chronic headache and anxiety disorder or depression.

The BMI was calculated by dividing the individual’s self-reported body weight (in kilograms) by the square of his or her height (in centimeters). Following the World Health Organization (WHO) definition, the BMI was categorized in four groups, 1) less than 18.5 (underweight), 2) 18.5-24.9 (normal weight), 3) 25–29.9 (overweight) and 4) ≥30 as obese (WHO 1995).

The impaired mental health status (subjective perceived psycho-social discomfort) was assessed with the questions “How often during the last four weeks did you feel”… “nervous?”, “abject?”, “sad?”, “exhausted?”, or “tired?”. Subjects were classified to suffer from psycho-social discomfort if at least one of these dimensions had occurred “always” or “mostly” within the preceding four weeks. Cronbach’s alpha of the so calculated indicator was 0.807, which shows a high internal consistency of the parameter.

### Statistical analysis

The data was analyzed using SPSS version 20 for Windows.

The descriptive analysis was conducted by means of cross-tabs. Differences between groups were assessed with the Pearson’s Chi^2^-test. In addition, a logistic regression model was used to analyze the association between having performed a preventive health check-up during the last three years (dependent variable) and age, socio-economic determinants, self-rated overall health status and satisfaction, presence of chronic diseases, and mental health status (independent variables). The results are presented as odds ratios with 95% confidence intervals.

All results were stratified by sex to account for differences of the type and number of tests offered to males and females.

### Ethical considerations

Secondary analysis of the AT-HIS 2006–07 was approved by the Ethics Committee of the Medical University Vienna (EC # 770/2011).

## Results

Of all participants, 2,870 men (41.6%) and 3,128 women (41.8%) had attended a comprehensive preventive health check-up within the three years preceding the survey. Table 1 shows participation rates in preventive health check-ups in male and female subpopulations.

**Table 1:** Proportion of men and women in various subgroups, who attended a comprehensive preventive health check-up during the past three years

	**Men (%)**	**P**	**Women (%)**	**P**
**Total**	41.6%		41.8%	
**Age **		<0.001		<0.001
(N = 2870/3128)				
15-40 yrs (N = 796/901)	31.2%		35.7%	
41-64 yrs (N = 1514/1531)	49.1%		48.9%	
65 yrs and older (N = 560/696)	44.5%		37.9%	
**Highest archived educational status**		<0.001		<0.001
Primary education (N = 353/826)	32.6%		36.2%	
Secondary education (N = 2157/1979)	42.3%		44.3%	
Tertiary education (N = 360/323)	50.6%		43.8%	
**Monthly net household income/household member**		<0.001		<0.001
Euro ≤ 600 (N = 880/1027)	35.8%		36.4%	
Euro 601–1500 (N = 1549/1754)	43.8%		45.0%	
>Euro 1500 (N = 435/340)	48.9%		46.9%	
**Country of birth**		<0.001		<0.001
Austria (N = 2705/2942)	43.9%		43.2%	
European Union 15 (N = 35/61)	26.3%		47.3%	
European Union 27 (N = 14/30)	18.9%		33.7%	
Former Yugoslavia and Turkey (N = 74/80)	20.1%		24%	
Others (N = 42/14)	27.6%		10.9%	
**Self-rated overall health status**		<0.001		0.005
Very good (N = 1011/1060)	40.0%		41.8%	
Good (N = 1213/1242)	44.5%		43.5%	
Moderate (524/642)	42.6%		40.6%	
Bad (N = 99/165)	29.9%		37.6%	
Very bad (N = 22/18)	29.3%		25.7%	
**Self-rated health satisfaction**		<0.001		<0.001
Satisfied (N = 2656/2825)	42.4%		42.5%	
Dissatisfied (N = 214/303)	33.9%		36.1%	
**Presence of chronic diseases**		<0.001		0.005
Yes (N = 1106/1354)	44.8%		43.7%	
No (N = 1764/1774)	39.9%		40.4%	
**Body Mass Index**		<0.001		0.633
Underweight (N = 10/89)	22.7%		41.0%	
Normal (N = 1123/1686)	39.0%		41.9%	
Overweight (N = 1385/951)	44.9%		42.5%	
Obesity (N = 352/403)	39.8%		40.1%	
**Subjective perceived psychosocial discomfort**		<0.001		0.002
Yes (N = 794/1129)	37.9%		39.5%	
No (N = 2075/1999)	43.3%		43.2%	

There was no difference in preventive health check-up utilization rates between the sexes.

Regarding socio-demographic variables, middle aged participants, participants with secondary education (women) or tertiary education (men), higher income and those, whose place of birth was Austria (men) or another member state of the EU-15 (women) were more likely to perform a preventive health check-up.

The proportion of male subjects who attended preventive health check-ups during the last three years varied between a maximum of 57.5% in those, aged 41–64, with tertiary educational level, a monthly net household income per household member of more than 1,500 Euro and born in Austria and a minimum of 0% in those, aged 15-40 years, with primary educational level, low income and born in Turkey or in former Yugoslavia. For female participants the attendance rates ranged between a maximum of 66.7% in those, aged 41-64, with secondary educational level, a monthly net household income per household member of more than 1,500 Euro and born in the EU-15, except Austria and a minimum of 25% in those, aged 15-40 years, with primary educational level, low income and born elsewhere.

Table 2 indicates the results of the multivariate logistic regression analysis, in which all independent variables were simultaneously included in the model. Factors that significantly predicted participation in preventive health check-ups were age between 41 and 64 years in both sexes, secondary educational level in women and secondary or tertiary educational level in men (in comparison to primary educational level), monthly net household income per household member higher than 600 Euros and being chronically diseased in both sexes.

**Table 2:** Influence of socio-demographic and health-related variables on the chance of participating in preventive health check-up; results of the multivariate logistic regression model

	**Men**			**Women**		
	**OR**	**95% CI**	**P**	**OR**	**95% CI**	**P**
**Age**						
**20-40 yrs**	1			1		
41-64 yrs	2.090.886	1.86-2.34	<0.001	1.66	1.48-1.86	<0.001
65 yrs and older	1.840.476	1.58-2.13	<0.001	1.1	0.96-1.27	0.187
**Highest achieved educational status**						
**Primary education**	1			1		
Secondary education	1.425	1.227-1.654	<0.001	1.280	1.138-1.439	<0.001
Tertiary education	1.884	1.523-2.330	<0.001	1.187	0.981-1.436	<0.001
**Monthly net household income/person**						
≤Euro 600.-	1			1		
Euro 601–1500.-	1.186	1.061-1.325	0.003	1.282	1.155-1.422	<0.001
>Euro 1500.-	1.356	1.149-1.599	<0.001	1.306	1.098-1.554	0.003
**Country of birth**						
Austria	1			1		
European Union 15	0.432	0.290-0.644	<0.001	1.072	0.752-1.528	0.701
European Union 27	0.359	0.197-0.655	0.001	0.737	0.471-1.154	0.182
Former Yugoslavia and Turkey	0.452	0.345-0.592	0.000	0.511	0.391-0.666	<0.001
Others	0.646	0.445-0.940	0.022	0.195	0.112-0.340	<0.001
**Self-rated overall health status**						
Very good	1			1		
Good	1.027	0.909-1.161	0.664	0.991	0.879-1.119	0.888
Satisfactory	0.892	0.746-1.067	0.213	0.983	0.831-1.164	0.846
Bad	0.568	0.412-0.783	0.001	0.976	0.730-1.305	0.870
Very bad	0.658	0.375-1.155	0.145	0.569	0.318-1.020	0.058
**Self-rated health satisfaction**	1.144	0.920-1.423	0.227	1.182	0.970-1.441	0.098
**Presence of chronic diseases (yes)**	1.206	1.070-1.360	0.002	1.185	1.059-1.326	0.003
**BMI**						
Normal	1			1		
Underweight	0.542	0.260-1.130	0.120	0.992	0.748-1.317	0.958
Overweight	1.111	0.996-1.240	0.059	1.016	0.909-1.137	0.779
Obesity	0.958	0.813-1.129	0.608	0.951	0.818-1.106	0.515
**Subjective perceived psychosocial discomfort (no)**	1.142	1.009-1.292	0.035	1.087	0.973-1.214	0.142

The highest odds ratios were found for the variables age and education: People aged 41 to 64 were approximately twice as likely to perform preventive health check-ups as younger people. Additionally, men equal or older than 65 years were also almost twice as likely to engage in health check-ups as men younger or equal than 40 years. Male participants with completion of tertiary education were nearly twice as likely to perform preventive health check-ups as those with primary education. For men, independent predictors additionally included absence of subjective perceived psychosocial discomfort and being born in Austria, rather than being born in another member state of the EU or elsewhere.

## Discussion

The main finding of the present investigation is that there is no sex specific difference in relation with health check-ups in Austria. Subjects, who do not report psychosocial discomfort or who feel satisfied with their health, perform health check-ups more often. On the other hand, people, who rate their health status as “good” or “moderate”, are more likely to attend health check-ups than those, who rate their general health status as “very good”. Middle-age, higher income, higher educational level, absence of migration background and the presence of a specific chronic disease stay significant predictors for participation in preventive health check-ups in men and women.

The present results show that universal access to health services is not automatically related to the elimination of structural and socio-demographic barriers. Performance rates of regular preventive health check-ups of 40% are not as high as aspired, even if they are generally higher than international utilization rates of preventive health services [10,11]. Even though more than 60% of the US population believes in the legitimacy of preventive health services [10], the use of such services only ranges around 20% per year and varies between region and insurance type [11]. In the evaluation of the 40% attendance rate at the Austrian health check-up it has to be taken into account that the campaign has been regularly offered for several years, instead of being a restricted unique offer. In view of the ongoing discussion on the beneficial impact of comprehensive preventive heath examinations on long-term health related outcome parameters [6], it has to be clarified whether specific health strategies are afflicted with the same barriers towards attendance as the comprehensive model. Whereas the usefulness of immunization programs is broadly accepted, concern on the value of specific health prevention services, such as wide scale diabetes screening has been raised one decade ago [12], but also recently [13], referring to elevated costs, and the fact, that long-term all-cause mortality rates did not differ between intervention and control groups. In this context, as international recommendations for preventive health services are continuously proved against state of the art and local practicability. A revision of the current version of the Austrian preventive health program, dating back to the year 2005 is planned for the near future and it is likely that the composition is going to be modified according to the current international evidence.

The absence of a gender difference in the attendance of regular health check-ups is particularly interesting in several viewpoints: on the one hand, previous data from Statistics Austria show that one decade ago, health check-up participation rates of the Austrian population were distinctly higher in women than in men [14]. On the other hand, the international literature describes higher utilization rates of both, preventive health examinations and therapeutic services, for women than for men [15,16]. One possible methodological explanation for this phenomenon might be differing definitions for preventive health check-ups, at times including gynecological cancer screening examinations, such as mammography screening and cervical cancer screening, which are otherwise excluded. An alternative explanation for today’s equal usage of preventive health care services in men and women might be that gender-specific interventions have changed the overall approach to illness and health [15].

The results of higher preventive health check-up attendance rates of middle-aged male and female participants in comparison to younger ones corroborate previous findings, describing a positive association between age and screening behavior for several diseases, such as colorectal cancer [17], cervical cancer [18] and breast cancer [19]. However, information on engagement in preventive health services of older people at the age of 65 and over is scarce. In our analysis, attendance rates for preventive health examinations in men and women above the age of 65 were lower than in middle-aged persons, but still higher than in those below 40 years. A similar inverse relationships between age above 65 and mammography screening behavior has been reported [20] and might be explained by the fact, that middle-aged people can be more easily reached by preventive health campaigns taking place e.g. at the work-place, while the present health care system is not primarily designed for the universal delivery of health promotion initiatives for the elderly. The reasons for lower participation rates in the youngest age group of survey participants remain to be elucidated. Younger people are likely to focus on other things than health-related issues, as they tend to take health for granted.

Suffering from at least one chronic disease or disorder was declared by forty percent of Austrian survey participants (more women than men). These participants engaged more in prevention, than people without such complaints. A major drawback of the present analysis is the cross-sectional study design, rendering it impossible to strictly distinguish between cause and effect within these findings. Several possible explanations might be proposed: (1) chronically diseased subjects, who are regularly in contact with health care entities and who are under permanent medical supervision, may be easily addressed and directed to preventive health check-ups, whereby reimbursement issues might contribute, at least in part. Alternatively, (2) the subjective desire to explore potential underlying medical reasons for complaints might increase the likelihood to attend a preventive health check-up, which is reasonable within the meaning of primary prevention and early detection of possible co-morbidities. This second hypothesis would explain, why those, who subjectively rate their health as only “good” or “moderate”, participate in health check-ups more often than those, who subjectively rate their health as “very good”. Lifestyle-related risk-factors, such as overweight and lack of physical exercise, are highly prevalent in the Austrian population [21,22] and are likely to account not only for the high prevalence of cardiovascular diseases, but also for chronic disorders of the musculoskeletal system, such as arthrosis, arthritis and rheumatism and chronic back pain [23-26]. In addition, the examination might serve not only as a preventive health instrument, but also as a counseling tool to help these patients with their complaints. Lastly, higher prevalence rates of chronic diseases in subjects, who engaged in prevention, might be attributed to the fact that conditions had been diagnosed during the health check-up and that higher “true” prevalence rates in people not attending cannot be excluded. From the present results it is therefore not possible to draw final conclusions on whether people attend a health check-up because of complaints or whether complaints are detected and/or explored as a result of attendance. In summary, it seems likely that the final effect of higher participation rates among chronically ill people is the result of a complex combination of several potential explanations.

Results of the present investigation suggest that persons satisfied with their health status, and persons without psychosocial discomfort might be more health conscious, as they are more likely to use the offer of preventive health services than their counterparts. In accordance, the German Socio-Economic Panel Study [27] and others [28,29] showed that a high level of health satisfaction encourages favorable lifestyle and prevention-oriented health behavior. The surprising finding of higher attendance rates in health check-ups in Austrian men and women, who rated their health as “good” or “moderate” in comparison to those, who rated it as “very good”, suggests that the preventive health check-up examination in its current form might be used by certain people as a tool to clarify health complaints, even if this type of examination has not primarily been intended for this. Preventive health check-ups, also named “health-examinations”, are not of an a priori diagnostic purpose, but focus on healthy people. Therefore, adaption of the program might be considered.

The fact that people with low socio-economic status are the most disadvantaged by the absence of a universal health care system is commonly known [30]. However, the present data demonstrates that even in countries like Austria, where health care is equally accessible to everyone independent of socio-economic status, inequalities in dimension and quality of delivery of primary preventive services still exist. In detail, a higher educational level obviously is associated with higher health literacy. Such a positive relationship between educational level and the utilization of certain preventive health care services already has been postulated twenty years ago [31]. It has been shown previously, that Austrians with migration background have different health behaviors, which could also influence the attendance rates of preventive health check-ups [22]. As it is very likely, that the number of Austrian inhabitants born abroad and/or do not have the Austrian nationality will rise within the near future, it is important to explore the causative mechanisms for their integration deficits with respect to education, career opportunity and also health outcome.

Data of the current analysis may be best interpreted within the meaning of the “prevention-theory” [31], signifying a positive influence of personal net income on health behavior, whereby high income not only gives financial security, but also facilitates the dedication of a person’s time resources to health-influencing benefits and to satisfy health requirements. In more detail, subjects with a high personal net income are more likely to pay attention to a healthy diet, to afford a comfortable accommodation and to go on vacation in order to relax.

Concern on the value of specific health prevention services, such as wide scale diabetes screening has been raised one decade ago [12], but also recently [13], referring to elevated costs, and the fact, that long-term all-cause mortality rates did not differ between intervention and control groups. In this context, the value of screening recommendations and programs has to be constantly judged against current scientific evidence. The different measures in the frame of the Austrian health check-up were designed and selected after careful and thorough review of current scientific evidence. However, the current version dates back to the year 2005 and a revision of the test program is planned for the near future. Regarding earlier [12] and ongoing controversies [13] on inclusion of specific screening services in comprehensive prevention check-ups, it is likely that the composition of the Austrian health check-up is going to be modified according to the current evidence, and it may be assumed, that especially diabetes screening tests are going to be eliminated.

### Strengths and limitations of the study

Strengths of the present investigation include the large sample size and the analytical design, which allowed adjusting for possible confounders. Usage of a comprehensive questionnaire and a consistent survey-interview-team increased the likelihood for high data consistency. In view of the large number and random selection of survey participants, a high external validity of results for Austria can be assumed. One major methodological limitation of the analysis is the fact, that data is cross-sectional and therefore is of limited explanatory power in relation to the influence of socio-economic parameters and other determinants on preventive health assessment performance. Furthermore, results are based on descriptive and self-reported survey data, rather than administrative data. There was no validation done using a medical chart or feedback of a physician. A methodological limitation of the analysis is that only persons from the age of 20 were included, as age was surveyed in 5 year interval-categories, starting with the category of 15 to 19 whereas the preventive health check-up examination is available for persons from the age of 19. As participants needed to recall services which they received up to three years ago, recall bias may have occurred. In addition, self reported data might be influenced by actual mood and timing. People, who recently have received a preventive health check-up, might be more likely to accept responding to a Health survey. As survey response bias therefore cannot be completely excluded, overestimation of the preventive health check-up adherence rate might have occurred [29].

## Conclusions

From the present results it can be concluded, that preventive health check-up utilization rates are relatively high in Austria, compared to other countries, but still not as high as intended and not equally distributed among subgroups. Personal, social and medical circumstances are key predictors for participation in preventive programs. These include in particular middle age, high socio-economic status and psychosocial comfort without migration background. On the other hand, a very high subjective health status in the absence of any chronic disorder represents a risk factor for the underuse of services to early detection of diseases. Transparency, but also public awareness of available preventive health services must be strengthened and information on the reasoning behind prevention should be better communicated. Most recently, the long-term benefit of periodic comprehensive preventive heath examinations on morbidity and mortality has been questioned by the US Preventive Service Task Force and the incorporation of specific individual preventive health services to targeted groups has been recommended instead [6].

Aspects, such as the thorough calculation of the benefit/harm ratio of the single screening components are going to be incorporated in the evaluation and revision processes of preventive health programs. Taking a population-wide perspective, prevention strategies will only succeed if participation rates for both, comprehensive and specific health services can be significantly augmented for subgroups with a documented underuse. Results of the present survey might help to improve the Austrian health care system by highlighting the fact that even with universal access to health services, structural and socio-demographic barriers remain to be overcome. One measure is the strengthening of the role of primary health care professionals. Austria has no gate keeping system which undermines the vital role family doctors can play for example by coordinating the services at the primary care level [32]. Another recommendation would be to further refine the information especially about specific preventive health services to the needs and the language of the target groups. Overall, the present study draws a positive light on the attendance of health check-ups in Austria, but highlights the need for a focused approach on key target groups.

## Consent

Written informed consent was obtained from the participants for the publication of this report and any accompanying images.

## Abbreviations

OECD: Organization for Economic Co-operation and Development; AT-HIS: Austrian health interview survey database; BMI: Body mass index; EU: European Union; WHO: World Health Organization.

## Competing interests

The authors declare that they have no competing interests.

## Authors’ contributions

The conceptualization and development of the framework for the manuscript was led by SBZ and TD. SBZ led the literature review and the writing of the paper. KG led the statistical analysis and wrote significant portions of the manuscript. VS wrote significant portions of the manuscript. All authors critically revised the manuscript and approved the final version.

## Pre-publication history

The pre-publication history for this paper can be accessed here:

http://www.biomedcentral.com/1471-2458/13/1138/prepub
